# The ability of teleost fishes to recognize individual faces suggests an early evolutionary origin in vertebrates

**DOI:** 10.3389/fpsyg.2024.1497386

**Published:** 2024-11-27

**Authors:** Masanori Kohda, Shumpei Sogawa, Will Sowersby

**Affiliations:** Laboratory of Animal Sociology, Department of Biology, Graduate School of Sciences, Osaka Metropolitan University, Osaka, Japan

**Keywords:** face recognition, face inversion effect, self-face recognition, representation, true individual recognition

## Abstract

The face is the most important area on the human body for visually differentiating between individuals. When encountering another person, humans initially gaze at and perceive the face holistically, utilizing *first-order* relational information and specific neural systems. Information such as identity and emotional state are then obtained from the face by distinguishing between small inter-individual differences, i.e., *second-order* relational information. Similar patterns and mechanisms underlying individual face recognition have been documented in primates, other social mammals, birds, and more recently in some fishes. Like humans, fish are capable of rapidly (<0.5 s) and accurately recognizing multiple familiar conspecifics by individual-specific variation in the face. Fish can also recognize faces from various distances and angles, providing evidence for mental representation of faces in this large and diverse vertebrate group. One species, the cleaner fish, has even demonstrated mirror self-recognition (MSR) via self-face recognition, strengthening the claim that non-human animals are capable of having mental images and concepts of faces. Here, we review the evidence for individual face recognition in fishes and speculate that face identification neural networks are both similar and widespread across vertebrates. Furthermore, we hypothesize that *first-and second-order* face recognition in vertebrates originated in bony fishes in the Paleozoic era ~450 Mya, when social systems first evolved, increasing the importance of individual recognition.

## 1 Introduction

More than any other part of the body the face plays a key role in human social interactions (e.g., Bruce and Young, 1998; Tanaka, 2001; Tanaka and Farah, 2003; Peterson and Rhodes, 2003). For example, human faces contain a range of important social cues that are exploited to assess conspecific identity, status, emotional state and intention (Keenan et al., 2003). Humans rapidly process and recognize faces in two stages, first by observing and perceiving the whole face using *first-order* information and then by using *second-order* individual-specific information to identify others, emotional states and intent (Civile et al., 2011; Rhodes, 2013).

The functions and processes underpinning face recognition in humans appear to be similar to primates, other mammals and birds (e.g., Kano and Tomonaga, 2009; Parr, 2011). Many non-human animals have now demonstrated an ability to rapidly and accurately perceive and recognize faces in a “face-specific” cognitive process, despite changes in the angle or even age of faces (e.g., Leopold and Rhodes, 2010). Unsurprisingly, the evolutionary origin of face-specific perception has received increasing attention in the literature (e.g., Parr, 2011) although how to interpret the evolution of various associated phenomena, including the face inversion effect and holistic face processing, remains controversial (Burke and Sulikowski, 2013).

The teleost fishes are the largest and most varied vertebrate group, containing thousands of species with a wide range of different life-histories (Sowersby et al., 2022), ecological niches (Sowersby et al., 2021) habitat preferences (Helfman et al., 2009), and cognitive abilities (reviewed in: Bshary et al., 2002; Bshary, 2011; Oliveira, 2013; Bshary and Brown, 2014; Brown, 2015; Sneddon and Brown 2020). Fishes have well developed visual capabilities (Rosa Salva et al., 2014) and over the last 15 years several studies have tested and documented the capacity for different fish species to visually recognize and distinguish between faces (e.g., Siebeck et al., 2010; Kohda et al., 2015; Wang and Takeuchi, 2017; Saeki et al., 2018; Kawasaka et al., 2019; Sogawa et al., 2023, 2024). However, broad reviews of the evolution of face processing across taxa (Tibbetts and Dale, 2007; Leopold and Rhodes, 2010) including those on holistic face processing in primates and other species (Parr, 2011; Burke and Sulikowski, 2013) have generally not considered teleost fishes or their important phylogenetic position for informing the evolution of these ancestral vertebrate traits.

This review paper aims to fill these gaps. Here, we review the literature on face recognition in teleost fishes that has accumulated over the last decade and a half and compare the function and mechanisms underpinning this ability with terrestrial vertebrates. We describe in detail the various methods used to investigate different elements of face recognition in fishes and broadly consider the evolutionary and functional importance of this cognitive ability. Important to note when we refer to fish or fishes throughout this review paper, we are specifically referring to species of bony fishes (Teleostei) rather than cartilaginous (Chondrichthyes) or jawless fishes (Agnatha).

## 2 Face-perception

Studies have typically utilized newborn infants or stimulus naïve individuals to examine whether behavioral patterns are innate or influenced by learning. For example, studies on the development of face perception in humans have found that infants (less than 1–5 days old) prefer to view face-like images compared to non-face objects (Johnston et al., 1991). Studies by Johnston et al. (1991) and others [reviewed in Rosa Salva et al. (2011)] provide support for the idea that humans have an innate ability to perceive the human face. While any effects of learning cannot be completely ruled out, it does appear that the human brain possess a template for perceiving facial structures that enables humans to easily separate faces from the visual background (Johnston et al., 1991).

Species of monkey and neonate bird also demonstrate a pre-existing ability to perceive faces. For instance, Japanese macaque (*Macaca fuscata*) show preferences for face stimuli over non-face stimuli even when they have been reared without previous exposure to faces (Sugita, 2008). Likewise, neonate chicks demonstrate a preference for observing faces and peck at the adult head more than other body parts (Johnston et al., 1991; Vallortigara et al., 2001; Rosa Salva et al., 2011; Kobylkov and Vallortigara, 2024). These studies across distantly related non-human vertebrate taxa imply an innate preference for observing face-like stimuli and an ability to perceive and recognize the importance of faces, even without previous experience.

In fishes, juveniles of a small reef species, the blue-green chromis (*Chromis viridis*), appear to be able to distinguish between different types of species by their face. For example, when juvenile chromis were presented with fish-face models representing a typical fish predator and a non-threatening algae feeder (Karplus and Algom, 1981) they tried to initiate an escape from the predator face model much sooner than from the non-predatory fish model (Karplus and Algom, 1981). The juvenile chromis likely used differences in face structure to assess risk, including total mouth size and eye size/position, which are typically accurate distinguishing features between predatory and non-predatory fish species (Karplus and Algom, 1981; Karplus et al., 1982). Elsewhere, predator-naïve juvenile African jewelfish (*Hemichromis bimaculatus*) pay more attention to models with two black spots that resemble forward-facing eyes, compared to models that do not (Coss, 1978; Coss and Tyler, 2023). The ability to perceive a face (and/or eyes) relies on *first-order* relational information, which can increase the likelihood of detecting potential predators or receiving resources from parents (Diamond and Carey, 1986; Civile et al., 2011). The available evidence suggests that fishes have an innate ability to perceive faces, but unlike in other taxa these results have instead largely been interpreted as being comparable to innate releasing mechanisms (I.R.M.; an innate neural network in the brain that responds to a specific stimulus and triggers a particular response; Tinbergen, 1951; Karplus and Algom, 1981, Karplus et al., 1982).

## 3 Observing the eyes and face

**Figure 1:**
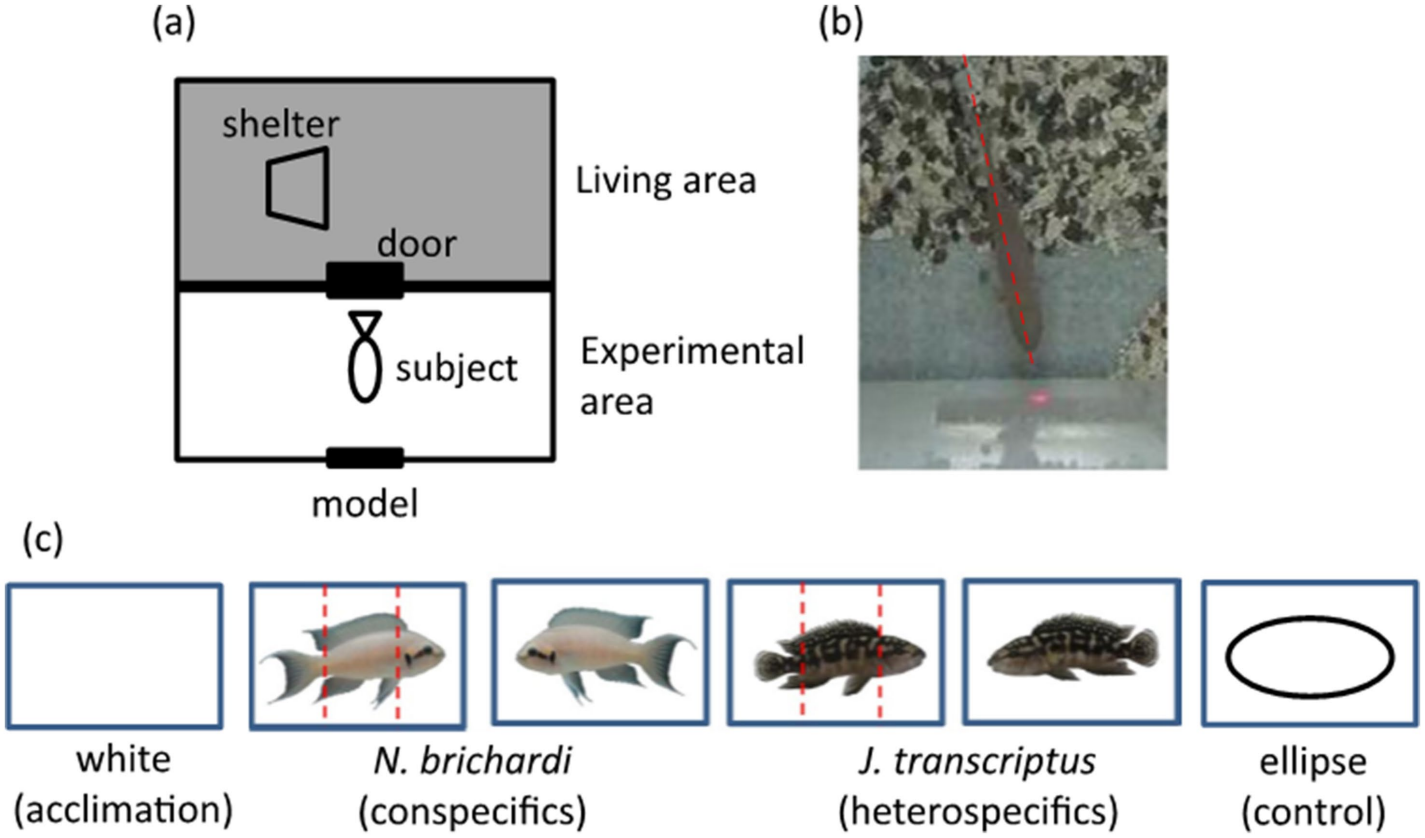
Experimental setup and stimulus image models used in Hotta et al. (2019). **(A)** Experimental setup. **(B)** Using body axis alignment to understand focus of visual attention. **(C)** Stimulus image models were mounted on cards: all cards were 4.3 cm wide. Hotta et al. (2019) identified which section of the card (see red dashed lines) was the focus of the focal fish’s gaze. Note that the eyes in this and many fish species are located on the side of the head. [Originally from Figure 1 in Hotta et al. (2019)].

Studies have shown that humans first gaze at another person’s face before observing other parts of the body (Johnston et al., 1991). The same behavioral pattern has been observed in other animals, with eye tracking observations showing that chimpanzees (*Pan troglodytes*; Hattori et al., 2010; Kano and Tomonaga, 2009, 2010), some monkeys (Parr and Heintz, 2008), and dogs (*Canis familiaris*) also gaze at the face before looking elsewhere on the body (Somppi et al., 2012). Until recently, the use of eye tracking or other methods to assess the focus of a fish’s visual attention have not been widely implemented. In 2019, Hotta and colleagues successfully exploited the direction along a fish’s body axis as a proxy for tracking its gaze (Figure 1). They found that daffodil cichlids (*Neolamprologus pulcher*) more frequently spend time focusing on the face, compared to the body or caudal areas, when they are first presented with images of both conspecific and heterospecific fish (Hotta et al., 2019).

Detailed eye-tracking observations in humans and primates have demonstrated that within the face the eyes are the initial focus of visual attention (Gothard et al., 2004; Kano and Tomonaga, 2009; Kano et al., 2012). The eyes also appear to be an important area of focus for fish, both for species recognition and to assess social status (Volpato et al., 2003; Karina et al., 2012). Indeed, Kawasaka (2021) found that the eyes are the most important feature that characterize a face in one fish species (i.e., *first-order* information). When Kawasaka presented daffodil cichlids with digitally altered images of conspecific faces they paid significantly less attention to images that were missing the eyes, compared to those with eyes or that had other features digitally removed (e.g., color markings or the mouth; Kawasaka, 2021). When combined with earlier face perception research in daffodil cichlids (Hotta et al., 2019), Kawasaka’s results provide compelling evidence that the eyes are a critical feature for visual perception of the face.

The position and orientation of eyes obviously differ across species (e.g., forward or profile facing) but nevertheless the eye appears to be the initial focus when animals, including fish, inspect and perceive a face (Karina et al., 2012; Kawasaka, 2021). Eye-like patterns are visual stimuli known to elicit social or predator response behaviors in fish, whether they appear on the bodies of other animals or have been placed on inanimate models (Coss, 1978; Coss and Tyler, 2023; Karplus and Algom, 1981; Karplus et al., 1982; Karenina et al., 2013). The young of many coral reef fish including butterflyfish, wrasse and damselfish often have a small false eyespot on the dorsal fin or broader dorsal area, while the real eyes are sometimes concealed by a black eye-line coloration (Fricke, 1976). Individuals are often observed with non-fatal injuries on the dorsal fin area (Neudecker, 1989) suggesting that predators associate eyes with prey, but can also be directed away from the head by the false eyespots (Neudecker, 1989; Kjernsmo and Merilaita, 2013). While previous studies on fish have investigated the potential primitive or pre-face recognition functions of eye detection (e.g., Emery, 2000) few studies have considered the complex cognitive processes involved in eye detection or face perception, including holistic processing or gaze following. Exceptions are the results from experiments on daffodil cichlids, which provide insights into the importance of the eye in the evolution of face perception (Hotta et al., 2019; Kawasaka, 2021).

## 4 Recognizing and identifying individual faces

The mechanisms animals use to identify other individuals has received much attention in the literature, especially among primatologists (Nahm et al., 1997; Gothard et al., 2004; Kano and Tomonaga, 2009, 2010; Kano et al., 2012). Humans, primates, several other mammals, and birds typically visually recognize and identify individuals via face recognition utilizing a “face-specific” cognitive process (e.g., Rosenfeld and Hoesen 1979; Kendrick et al. 2001; Tibbetts and Dale, 2007; Leopold and Rhodes, 2010) although olfactory cues (e.g., in mammals) and auditory cues (e.g., in birds) can also play a role in individual recognition (Leopold and Rhodes, 2010). Like humans, many other vertebrate species have demonstrated a remarkable ability to rapidly and accurately process and recognize individual faces (Kano and Tomonaga, 2009, 2010; Kano et al., 2012).

**Figure 2:**
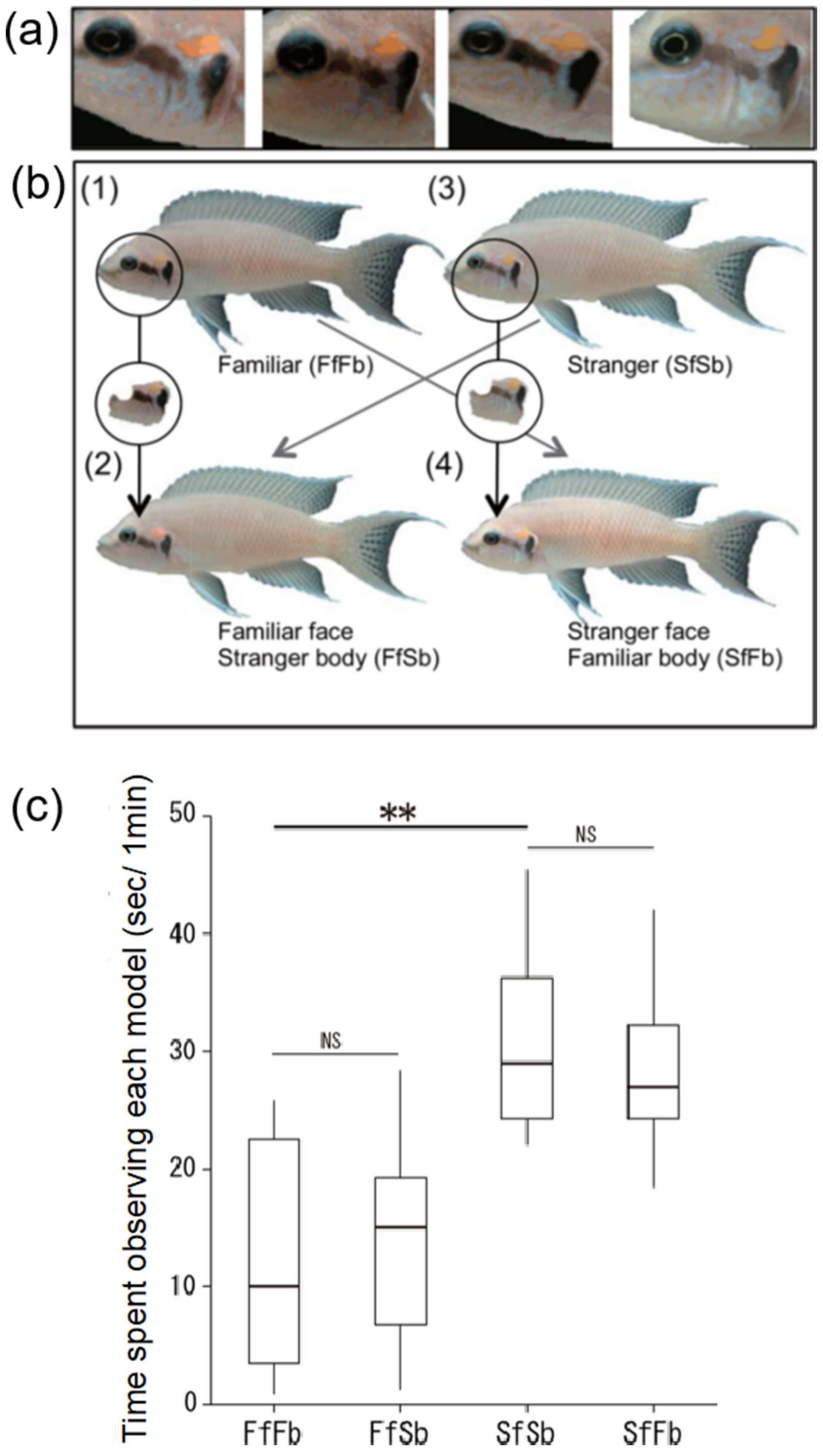
Distinctive facial color patterns and four types of images models presented to *Neolamprologus pulcher* in Kohda et al. (2015). **(A)** Individual variation in facial color pattern in four individuals. **(B)** Examples of the four different types of image models; FfFb: familiar neighbor, FfSb: familiar neighbor’s face and unfamiliar stranger’s body, SfSb: unfamiliar stranger, and SfFb: unfamiliar stranger’s face and familiar neighbor’s body. **(C)** Time spent observing the different model types. Median, box (showing 25 and 75%) and range. **p < 0.01, NS *p* > 0.05 (Wilcoxon signed rank test). Originally from Figures 1A,B in Kohda et al. (2015).

The adaptive significance of individual face recognition is thought to be its benefit for social interactions. Sedentary fish species often live in highly structured and complex social groups. For example, in tropical lakes and coral reefs many sedentary fish species live in monogamous pairs, family groups with related/unrelated brood helpers, or in harem polygamy with associated dominance orders and territory defense (Thresher, 1984; Gross and Sargent, 1985; Taborsky, 1994; Kohda, 1997; Kuwamura, 1997; Awata et al., 2005). In these societies, individual recognition is essential for maintaining social interactions and behaving appropriately toward familiar and unfamiliar conspecifics (Bshary, 2011; Bshary et al., 2014). Beginning with the daffodil cichlid (Figure 2) individual identification via face recognition has now been observed in multiple social fish species, which are all capable of forming stable and long-term social relationships (e.g., Siebeck et al., 2010; Kohda et al., 2015; Satoh et al., 2016; Hotta et al., 2017; Sogawa et al., 2023, 2024).

Daffodil cichlids have individual-specific color variation on the face, and it has been hypothesized that this feature is used to distinguish between individuals (Figure 2A; Kohda et al., 2015). The daffodil cichlid is also highly territorial and acts with aggression toward unknown conspecifics while remaining tolerant toward familiar conspecific neighbors (Frostman and Sherman, 2004; Sogawa et al., 2016). The territorial behavior of the daffodil cichlid has been exploited to experimentally test its ability to distinguish between familiar and unfamiliar faces. Specifically, Kohda et al. (2015) presented four different types of model images (photographs) to captive daffodil cichlids, consisting of (i) a familiar neighbor, (ii) an unfamiliar individual, and two digitally altered composite image models of (iii) a familiar neighbor’s face on an unfamiliar individual’s body, and (iv) an unfamiliar individual’s face on a familiar neighbor’s body (Figure 2B). The image models with the familiar neighbor’s face, regardless of whether it was with the correct or on an unfamiliar body, were observed less frequently compared to the unfamiliar individual’s face, indicating that daffodil cichlids can distinguish between faces independently of the body (Figure 2C; Kohda et al., 2015). The use of images, including digitally altered composite images, has recently become a standard method for testing face recognition abilities in fishes, including between familiar neighbors and unfamiliar individuals (e.g., Satoh et al., 2016; Hotta et al., 2017; Sogawa et al., 2023) and between the self (in a mirror) and familiar fish (Kohda et al., 2023). The presentation and alteration of images offers an interesting avenue for future research on how fish process *second-order* relational information for example, to uncover which color signals are important for identifying individual faces.

While daffodil cichlids use distinct color markings to identify individuals, it remains unclear whether the position of these markings on the face is critical for individual identification (Kohda et al., 2015). Interestingly, other fish species such as discus (*Symphysodon* sp.; Satoh et al., 2016) and the golden julie (*Julidochromis ornatus*; Hotta et al., 2017) have distinct color markings over the whole body, yet studies have demonstrated the central importance of the facial area for individual recognition in these species. For example, when presented with four different types of image models (similar methodology to Kohda et al., 2015) both species act less aggressively toward models with a familiar conspecific face, independent of whether the body in the image is from a familiar or unfamiliar individual (Satoh et al., 2016; Hotta et al., 2017). Both discus and golden julie have individual-specific coloration on the face and the body, but only the face appears to be important for distinguishing between familiar and unfamiliar individuals. Discus and golden julie are social species, with discus forming reproductive pairs and golden julie employing cooperative polyandry, and both can visually recognize partners and group members (Satoh et al., 2016; Awata et al., 2005).

The fish examples discussed in this review thus far have largely been from the one family, the Cichlidae. But it is becoming increasingly clear that face recognition is phylogenetically widespread across teleost fishes. For instance, evidence for face recognition has now been found in 10 species from 7 families (representing four Orders) including the Pomacentridae (Siebeck et al., 2010), the Labridae (Kohda et al., 2023), the Gasterosteidae (*Gasterosteus aculeatus;* Sogawa et al., 2024), the Adrianichthyidae (*Oryzias latipes*; Wang and Takeuchi, 2017) and the Poeciliidae (*Poecilia reticulata*; Sogawa et al., 2023; Table 1). Common features of the species that have demonstrated face recognition is living in stable social systems and having individual-specific color patterns on the face, typically on the operculum or cheek, nearby the eye (Table 1). Given the tendency for vertebrates including fish to focus on the eye when first observing other individuals (Coss, 1978; Coss and Tyler, 2023; Karplus et al., 1982; Kawasaka, 2021) we hypothesize that individual-specific color patterns nearby the eye allow for rapid individual recognition in social fishes. Numerous species that have not been tested for face recognition ability, including other wrasse species (Labroidae) and the marine angelfishes (Pomacanthidae), are also highly social (e.g., harem polygyny; Kuwamura, 1984; Thresher, 1984; Sakai and Kohda, 1997) and appear to have individual variation in face color patterns [e.g., see Masuda et al. (1984)]. We therefore suggest that these and many other social species of teleost fish can likely perceive faces and recognize other individuals via face recognition.

**Table 1:** Species of fish represented in face recognition experiments.

Reference	Order/ Family	Species	Capable of individual face recognition	Individual-specific color patterns on the operculum	Social system	Habitat
Perciformes						
[1]	Cichlidae	Daffodil cichlid	Yes*	Yes	Cooperative breeding	Lake Tanganyika
[2]		Golden julie	Yes	Yes	Cooperative breeding	Lake Tanganyika
[3]		Discus	Yes	Yes	Sexual pair	Amazon rivers
[4]		Jewelfish	Yes*	Yes	Sexual pair	West African rivers
[5]	Pomacentridae	Ambon damsel	Yes	Yes	Territorial	Coral reef
[6]	Labridae	Cleaner wrasse	Yes	Yes	Harem polygyny	Coral reef
Gasterosteiformes						
[7]	Gasterosteidae	Three-spined stickleback	Yes	Yes	Territorial	High altitude rivers
[8]	Syngnathidae	Messmate pipefish	Yes^+^	Yes	Sexual pair	Coastal areas
Beloniformes						
[9]	Adrianichythydae	Medaka	Yes	Yes	Dominance/territorial	Rivers and ponds
Cyprinodontiformes						
[10]	Poecillidae	Guppy	Yes	Yes	Dominance/territorial	Rivers

Reference: [1] Kohda et al. (2015); [2] Hotta et al. (2017); [3] Satoh et al. (2016); [4] Coss and Tyler (2023); [5] Siebeck et al. (2010); [6] Kohda et al. (2023); [7] Sogawa et al. (2024); [8] Sogabe (2011); [9] Wang and Takeuchi (2017); [10] Sogawa et al. (2023).

*Individual variation in face color patterns are experimentally shown to be the signal exploited for individual recognition.

^+^Individual variation in face color patterns not tested but suggested by the authors as an important signal.

Kohda et al. (2015) not only demonstrated that the daffodil cichlid is capable of face recognition, but also found that they can accurately and rapidly (<0.5 s) discriminate between familiar and unfamiliar faces, comparable to primates and humans. The ability to rapidly and accurately identify individuals and to be recognized by others has obvious advantages for social species in which individuals often interact. The importance of individual identification in social species is likely acting as a strong selective pressure driving face recognition and individual variation in markings and color patterns (Tibbetts and Dale, 2007). Moreover, the location of individual-specific signals close to the eyes has potentially evolved to facilitate rapid signaling given the important role the eye plays in face perception. The prominent role of the face does not mean that important signals and cues are not also obtained from the body. Signals alluding to the physical condition of an individual, important in mate choice or rival assessment are obtained from the body; e.g. bright red coloration on the belly of three-spine sticklebacks (Candlin, 1999), orange spot on the flanks of the guppy (Endler, 1995), or the white patch on the frontal trunk of the bluehead wrasse *Thalassoma bifasciatum* (Warner and Schultz, 1992).

## 5 True individual recognition (TIR) via the face

In social animals the ability to identify others is essential for maintaining social interactions (Tibbetts and Dale, 2007; Tibbetts, 2002). Visual identification of others by face recognition has been particularly well documented in primates and some other non-primate social mammals (Leopold and Rhodes, 2010; Parr, 2011). The ability of an animal to accurately identify multiple individuals is regarded as ‘true individual recognition’ (TIR; Tibbetts and Dale, 2007).

We have highlighted several examples of fish species that can rapidly and accurately distinguish between the faces of familiar and unknown individuals. The ability to distinguish between different types of individuals and place them into different categories is known as ‘class level recognition’ (CLR) but does not represent TIR (Tibbetts and Dale, 2007; Parker et al., 2020). Achieving CLR requires signals to differentiate between categories of individual (e.g., between color morphs, phenotypic differences between male and female, or familiar and unfamiliar individuals; Tibbetts and Dale, 2007). Several studies have revealed that fish are capable of distinguishing between familiar and unfamiliar individuals, suggesting they are capable of CLR. For instance, daffodil cichlids can distinguish between familiar neighbors and strangers via face recognition (Kohda et al., 2015). However, the daffodil cichlid can also recognise individuals via individual-specific face color patterns, implying they are also capable of TIR (Saeki et al., 2018). We therefore speculate that if the methods used in Saeki et al. are replicated in other fish species, then many other species with individual-specific face patterns will demonstrate TIR. True individual recognition and CLR are often confused in the literature, yet in social animals it is TIR that is necessary for maintaining effective social interactions (Bshary, 2011; Bshary et al., 2014).

The daffodil cichlid is the first fish species to demonstrate TIR under experimental conditions (Saeki et al., 2018). Saeki and colleagues found that daffodil cichlids accurately distinguish between the faces of two familiar neighbors, i.e., between two individuals in the same class level. Very recent experimental evidence suggests that three-spined stickleback are also capable of TIR (Sogawa et al., 2024). Sticklebacks like many other fish species often maintain territories adjacent to the territories of other individuals (Mori, 1993). Under these spatial arrangements, territory holders appear to recognize other individual territory holders (e.g., Kohda, 1997; Itzkowitz and Leiser, 1999) with TIR potentially allowing the forming of so-called dear enemy relationships (Fisher, 1954; Ydenberg et al., 1988; Temeles, 1994). We hypothesize that comparable TIR mechanisms also help maintain social relationships in fish species with dominance hierarchies and sexual pair bonds. We consider it plausible that in social fishes where individuals repeatedly interact that TIR is established via individual face recognition. Our hypotheses extend beyond the teleost fishes and include other social vertebrates that visually identify familiar individuals.

In humans, visual TIR (true individual recognition) of multiple familiar people requires a mental image or concept of others, including their face (Keenan et al., 2003). For instance, humans can recognize familiar faces at different angles, not because of an internal face template, but by referencing a mental representation (or concept) of the face (Parr, 2011). Long-tailed macaques also recognize individual faces from different angles (Dasser, 1987) suggesting they to reference mental representation of faces. We consider it likely that other social vertebrate species capable of TIR also have mental images (representation) of familiar faces, rather than a simple internal template and that this fundamental ability has been conserved rather than repeatedly evolved across vertebrate taxa.

Observing and recognizing faces is more difficult in the wild compared to under laboratory conditions. In the wild other individuals are moving, they appear at various directions and angles, and they may be close or far away. Therefore, to recognize individual faces in natural conditions fish require robust mechanisms facilitating face recognition (Leopold and Rhodes, 2010). Remarkably, in 2018 Newport and colleagues described the ability of archerfish (*Toxotes* sp.) to recognize human faces. The archerfish were able to identify images of human faces even when the images were rotated and at different angles, leading to the suggestion that archerfish exhibit some degree of spontaneous view generalization (Newport et al., 2018). Utilizing internal templates to recognize individual faces in wild situations requires learning and recall not only of the individuals, but also templates of those individuals when viewed from different angles. An explanation of view generalization would allow fish to generate numerous face patterns from a limited number of templates. However, if the mechanisms underlying face recognition in fish are like humans, then Newport et al.’s (2018) results could also be explained by archerfish having mental representations (i.e., concept) of human faces rather than via view generalization of face templates. We consider the mental representation a more parsimonious and inclusive explanation, given the inherent advantages of having flexible mental images for identifying multiple individuals at different angels in the wild (e.g., Leopold and Rhodes, 2010). We discuss this idea further in the next section using two recent experimental examples (Kohda et al., 2023).

## 6 Self-face recognition plays a key role in mirror self-recognition

**Figure 3:**
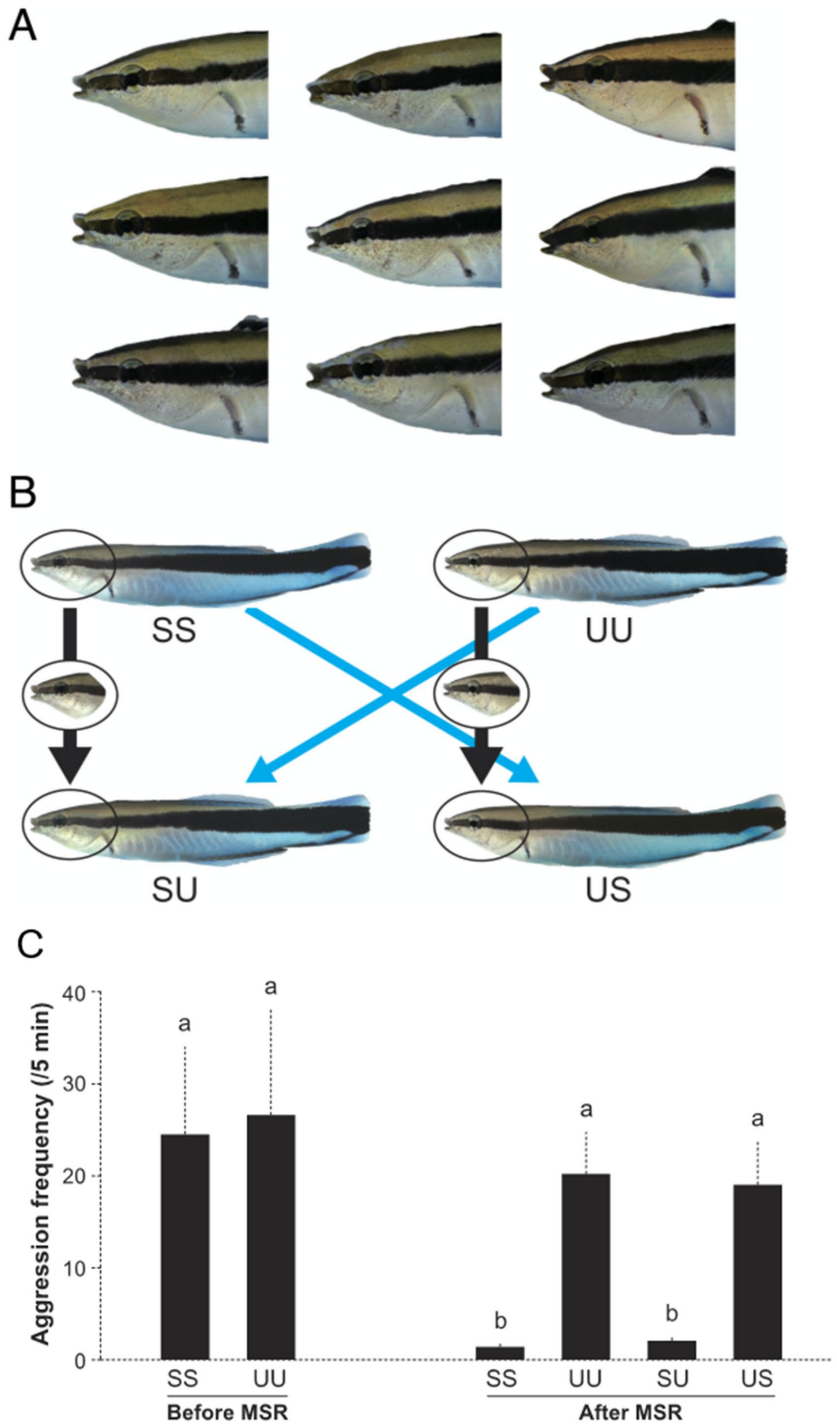
Cleaner fish image models (photographs) used in Kohda et al. (2023). **(A)** Faces of focal fish. **(B)** Examples of image models used in the experiment; SS, self-model, UU, unfamiliar fish model, SU: self-face/unfamiliar body model, US, and unfamiliar face/self-body model. **(C)** Frequency of aggressive behavior directed toward image models. Before MSR = before mirror presentation, after MSR = after passing the mark test. Mean aggression (SEM), Friedman test, χ_5_2 = 36.51, *n* = 10, *p* < 0.0001, and Kendall W (effect size) = 0.730. a and b represent statistically significant differences by exact Wilcoxon signed-rank tests with sequential Bonferroni adjustments. Originally from Figures 2 and 3A in Kohda et al. (2023).

Humans identify the self in a mirror reflection (their self-image) via self-face recognition (Keenan et al., 2003). Specifically, humans appear to recognize the self-face in the mirror (or a photograph) by referencing a memorized mental representation of the self-face. Other species are capable of mirror self-recognition (MSR), including chimps (Gallup, 1970), dolphins (*Tursiops* sp.; Reiss and Marino, 2001), Asian elephant (*Elephas maximus*; Plotnik et al., 2006), Eurasian magpies (*Pica pica*; Prior et al., 2008) and the house crow (*Corvus splendens*; Buniyaadi et al., 2020). It has remained unclear and somewhat controversial exactly what features and mechanisms non-human animals use to recognize themselves in a mirror. However, recently Kohda et al. (2023) have provided compelling experimental evidence using cleaner fish (*Labroides dimidiatus*) to demonstrate how animals recognize the self in an image (Figure 3). Under laboratory settings, Kohda and team presented mirror-naïve cleaner fish with photograph models of unfamiliar (i.e., stranger) conspecifics and a model of the self. Focal fish acted aggressively toward both the unfamiliar individual and self-image models (Figure 3C). After exposure to a mirror and undergoing and passing the mirror mark test (see Kohda et al., 2019) focal fish then exhibited significantly less aggression toward the self-image, compared to the photograph of the unfamiliar individual. When subsequently presented with digitally altered composite models, focal fish exhibited similar levels of aggression toward models composing of an unfamiliar face with the self-body as they did toward complete unfamiliar individual models. Moreover, focal fish displayed significantly less aggression toward composite models of the self-face with an unfamiliar body (Figure 3C). Kohda et al.’s (2023) results clearly demonstrate that after mirror exposure, images of the self-face are recognized as the self and subsequently elicit significantly less aggression.

Kohda et al. (2023) suggest that self-face recognition is only possible after cleaner fish have been exposed to the self-image in a mirror. However, there remains the possibility that cleaner fish are not recognizing the self but instead consider the mirror reflection and consequently the self-image model to be a now familiar individual (via true individual recognition). To exclude this possibility Kohda et al. also presented cleaner fish that had previously passed the mirror mark test with self-images that had a mark digitally placed on the throat area of the image. The focal fish reacted by scraping their own throat on available substrate, which is the same behavioral response cleaner fish exhibit when viewing their mirror reflection when an actual mark has been placed on their throat (e.g., see Kohda et al., 2019, 2022). The behavioral response to the marked self-images implies that cleaner fish can recognize the mirror and other images as the self and do so via self-face recognition.

An alternative explanation for cleaner fish mirror self-recognition (MSR) is that cleaner fish recognize the self via kinesthetic visual matching, rather than by recognizing the self-face. Like in a mirror reflection, humans identify themselves and other familiar people in photographs using mental representations (i.e., concept) of the self and of others (Keenan et al., 2003). Kohda et al. (2023) found that cleaner fish can also recognize the self in photographs. Because photographs are motionless, recognizing oneself in a photograph cannot be done via kinesthetic visual matching, but by referencing a mental representation or concept of the self, originally obtained from observing the self-image in a mirror (Kohda et al., 2023). We speculate that in cleaner fish recognition of the self-face during MSR involves the same mental processes involved in the true individual recognition of familiar individuals. Furthermore, we predict that other species that demonstrate MSR will also be capable of photograph self-recognition by recognizing and referencing mental representations of the self-face (Kohda et al., 2023).

How can an animal obtain a mental representation of the self-face? In cleaner fish, we hypothesize that a mental representation of the self-face develops during mirror exposure and is incorporated into the individuals pre-existing sense of the self (Kohda et al., 2023). Animals are unlikely to encounter a mirror in the wild, so our hypothesis is that the mental processes that allow MSR and photograph self-recognition under experimental conditions have evolved in nature for TIR of conspecifics. Furthermore, the available evidence implies that the way cleaner fish and humans process MSR and photograph self-recognition is similar and that both species have a concept of the self and of others (Keenan et al., 2003; Kohda et al., 2023; Kobayashi et al., 2024).

## 7 The face inversion effect

Humans can more easily and rapidly recognize faces compared to other objects or visual patterns (Bruce and Young, 1998; Tanaka, 2001; Peterson and Rhodes, 2003). From the face, humans are also able to quickly recognize other people’s emotional states and the direction of their gaze and attention. Face recognition is possible due to face-specific cognitive and neural mechanisms, which process the face holistically, rather than featurally like other non-face objects (Tanaka and Farah, 2003). The face inversion effect (Yin, 1969) describes the phenomena where an inverted face disrupts configural (holistic) processing and impacts the ability of humans to perceive and recognize upside down faces, compared to other objects (Valentine, 1988, but see Racca et al., 2010). The existence of the face inversion effect provides compelling evidence for holistic face processing in humans and has proven to be important in our understanding of the evolution of face recognition mechanisms (Civile et al., 2016).

The evolutionary origin of face-specific perception has understandably generated much research interest (Parr, 2011). Yet despite the face inversion effect occurring in other animals, the ability of non-human animals to holistically process faces has remained controversial (Burke and Sulikowski, 2013). Chimps (Parr et al., 1999; Tomonaga, 1999; Parr et al., 2000; Tate et al., 2006), spider monkeys (*Ateles* sp.), rhesus macaque (*Macaca mulatta*; Parr, 2011), sheep (*Ovis aries*) and the budgerigar (*Melopsittacus undulatus*) are examples of other animals that have demonstrated the face inversion effect (Brown and Dooling, 1992, 1993; Kendrick et al., 1995). However, certain species, including crows and some monkeys so far have not (Parr, 2011; Brecht et al., 2017). Why some species but not others have trouble identifying upside-down compared to upright faces remains unclear, but taxa appropriate methodological issues inducing false negative effects cannot be excluded (Racca et al., 2010; Parr, 2011; Kohda et al., 2022). False negatives are not uncommon in behavioral experiments and may provide an explanation for why some species do not demonstrate the face-inversion effect (e.g., Beckoff and Sharman, 2004; Rajala et al., 2010; Kohda et al., 2022).

The face inversion effect has also been demonstrated in two phylogenetically distant fish species, the medaka (*Oryzias latipes*) and the daffodil cichlid (Wang and Takeuchi, 2017; Kawasaka et al., 2019). Medaka take longer to distinguish between familiar and unfamiliar fish when faces are inverted but can readily identify other upside-down non-face objects (Wang and Takeuchi, 2017). Similarly, when faces are upright daffodil cichlids spend more time watching unfamiliar faces, however when faces are inverted there is no difference in the time spent watching familiar or unfamiliar faces, indicating that the face inversion effect is occurring (Kawasaka et al., 2019). These two studies both suggest that these fish visually perceive and process faces holistically, potentially using comparable mechanisms to humans and other vertebrates. The existence of the face inversion effect in two phylogenetically distant fish species suggests that this phenomenon is likely to be present in other fishes capable of face recognition (Kawasaka et al., 2019).

As the face inversion effect was first detected in humans and then primates and other mammals, it was assumed that a large complex brain and associated neural networks were required to process faces holistically (Parr, 2011; Burke and Sulikowski, 2013). However, we suggest that the presence of the face inversion effect in fishes (Wang and Takeuchi, 2017; Kawasaka et al., 2019) demonstrates that a large brain is not a prerequisite for configural processing and viewing faces holistically. Indeed, evidence is now suggesting that invertebrates, such as bees and wasps also view and perceive faces holistically (Avarguès-Weber et al., 2018).

## 8 Identifying emotional state and direction of attention from the face

Human faces are informative visual stimuli important for perceiving emotional states and intention (Schmidt and Cohn, 2001). In humans and other mammals, facial expressions and movements are primarily produced by the action of muscles beneath the skin (Rinn, 1984; Tate et al., 2006; Leopold and Rhodes, 2010; Cattaneo and Pavesi, 2014). Comparable elaborated facial musculature is lacking in fishes and emotional facial expressions appear to be largely restricted to mammal taxa (Leopold and Rhodes, 2010).

Several fish species, including the daffodil cichlid and discus, can alter the appearance of facial color patterns to convey information on social dominance and fighting motivation (Balzarini et al., 2017; Satoh et al., 2024). These social or emotional signals are however not restricted to the face in fish and can also occur elsewhere on the body (Baerends and Baerends-van Roon, 1950; Plate I in Eibl-Eibesfeldt, 1974). Unlike mammals, the ability of fish to perceive emotional states from facial expressions seems limited (Leopold and Rhodes, 2010; Satoh et al., 2024) although admittedly very few studies have investigated this ability. As we have outlined in this review, face perception, with a particular focus initially on the eyes (*first-order* relational information) is observable in many fish species. Likewise, the ability to process faces holistically and to rapidly and accurately recognize individuals via small individual-specific facial differences (*second-order* relational information) is reported in several species (e.g., Kohda et al., 2015; Wong and Takeuchi Betancur-R et al., 2017; Kawasaka et al., 2019). Interestingly, Satoh and colleagues recently suggested that the ability to exhibit and perceive emotional facial expressions likely evolved later in vertebrates, with the mammals (Satoh et al., 2024).

Faces allow an observer to understand the direction of another individual’s gaze and attention (e.g., Kobayashi and Kohshima, 1997; Emery, 2000). For instance, many non-human animals can exploit faces to understand social cues, including dogs, goats (*Capra hircus*), jackdaws (*Coloeus* sp.) and crows (Zeiträg et al., 2022; Wilson et al., 2023). Experimental evidence has demonstrated that archerfish can use social cues such as conspecific orientation to rapidly predict the location of visual targets (Leadner et al., 2021). It has been suggested that social cues, including gaze, face and body orientation, may have an early evolutionary origin and can elicit automatic shifts of attention in observers [reviewed in Zeiträg et al. (2022)]. The importance of eye gazing and face orientation as social attentional cues in fishes has received limited attention in the literature, however we speculate that the social aspects of shared attention may be widespread in fishes.

## 9 The evolutionary origins of vertebrate face recognition: social systems and visual neural systems

The social behaviors of fishes, particularly in regard to reproduction and social structures, have been intensively studied in the wild over the last four decades (e.g., Thresher, 1984; Kuwamura, 1984, 1997; Kohda, 1997). Like social mammals and birds, many sedentary fish species exhibit parental care as part of nesting (bi-parental and uni-parental; Blumer, 1982; Barlow, 1984; Gross and Sargent, 1985; Clutton-Brock, 1991) and mating strategies (Davies et al., 2012), which is often associated with dominance hierarchies and territory maintenance (e.g., Thresher, 1984; Taborsky, 1984, 1994; Kuwamura, 1997). We hypothesize that sedentary teleost fish in freshwater and coastal areas in the Paleozoic era had likely developed complex social interactions and that selection pressures existed for the evolution of individual face recognition. We speculate this may have occurred as early as 450 mya considering that is when the visual neural systems now underlying face recognition first evolved (Betancur-R et al., 2017). The cognitive abilities and mechanisms underpinning individual face recognition therefore likely evolved during the early stages of social evolution, when the *second-order* relational information by which faces differ became important to process and recognize.

The exploitation of *first-and second-order* relational information and the disruption of holistic face processing demonstrated by the face inversion effect support the claim that face recognition occurs via specific neural systems, which differ from other visual neural systems (Bernstein and Yovel, 2015; Civile et al., 2016). The likely latest origin of face recognition, including the necessary neural mechanisms, would be with a teleost ancestor in the Devonian period. Considering that individual recognition via face recognition exists in social interactions from fish to primates, this important cognitive ability vital for social interactions has therefore been conserved in social species throughout evolutionary history. Given the available evidence, we consider it unlikely that face recognition and associated underlying neural mechanisms have independently evolved in subsequent vertebrate linages (e.g., birds and mammals) but have instead remained largely conserved across vertebrates.

More broadly, the function of neural systems in the brain associated with social decision-making has been conserved across vertebrates, including in fishes (O'Connell and Hofmann, 2012; Bshary et al., 2014; Ogawa et al., 2021). The current neural model for face recognition suggests a division of labor between the fusiform face area (FFA), which processes static facial aspects (e.g., identity) and the posterior superior temporal sulcus (pSTS), which processes more changeable facial aspects (e.g., expression; Haxby et al., 2000; Kanwishier and Yovel, 2006; Gobbini and Haxby, 2007). While primary visual sensory areas may be considered homologs across vertebrates (Yamamoto and Ito, 2002), the neural systems associated with face recognition have not yet been subject to detailed investigation in teleost fishes. We speculate, however, that it is unlikely that specialized neural systems for face recognition associated with mental representation of faces would evolve independently in different vertebrate groups. Thus, the most plausible evolutionary origin for the fundamental neural mechanisms involved in face perception/recognition in vertebrate taxa such as birds and mammals is with a common ancestor from the Devonian period.

## 10 Concluding remarks

Face naïve fish have an innate ability to perceive faces, with the eyes appearing to be the most prominent facial feature. In vertebrates, evidence suggests that faces are processed holistically, using *first-order* relational information and utilizing specific neural systems.

Like mammals, fish initially and repeatedly gaze at the face when encountering an individual. Several animal species are known to have the ability to recognize individuals via the face, including social fish species. This ability to perceive faces and recognize individual faces appears phylogenetically widespread in teleost fishes, occurring across several families and at least four taxonomic orders (Table 1).

In the wild, identifying individuals by their face can be challenging, with faces appearing at different angles and distances. Nevertheless, fish have demonstrated a remarkable ability to rapidly perceive and identify faces at different angles. To do so requires a degree of flexibility only achievable via referring to mental representations of faces, not learned fixed internal templates. The fact that fish can also recognize the self in photographs, ruling out kinesthetic visual matching, provides additional support for this claim.

Fish recognize the face holistically and use configural processing to rapidly identify faces. We know this because like humans and other mammals, fish exhibit the face inversion effect, where upside down faces are harder to recognize compared to upright faces. The small differences between faces, *second-order* relational information, are exploited to accurately recognize individuals.

Unlike humans, primates, and other mammals, fish do not appear to use faces to express and understand emotional states. Some fish species can alter color patterns to reflect emotional changes, but this is not restricted to the face and can occur elsewhere on the body. Fish can recognize and respond to social cues such as gaze and face direction to extract socially relevant information. However, in general, the ability of fish to express and perceive emotional states via the face and to respond to facial social cues remains relatively understudied compared to other vertebrate taxa and is an avenue for further research.

As our review has detailed, how fish perceive and recognize individual faces is more comparable to humans and other mammals than previously appreciated. This is despite fishes having relatively smaller brain sizes and often being considered less capable of complex cognitive abilities. Many fish species do however live in complex social structures, where being able to rapidly and accurately visually identify individuals is particularly important. Considering the similarities in the visual neural system across vertebrates, we propose that the cognitive abilities facilitating individual face recognition originated in bony fish no later than the Devonian period (Zimmer, 1998).

For decades the ability of animals to recognize faces was interpretated as instinct and/or associative learning [e.g., the triune brain hypothesis; MacLean (1967)]. A top-down anthropocentric view was largely accepted, whereby only humans were cognitively capable of having mental representations of faces. Yet, recent detailed experiments and investigations have highlighted the similarities in brain structure and neural systems across vertebrates, including teleost fishes (O'Connell and Hofmann, 2012) and have provided compelling evidence that non-human vertebrates such as fish do reference mental representation of faces (Kohda et al., 2023). Using humans as the focal point of comparison in evolutionary studies can hinder our progress and we recommend a bottom-up approach to understanding the evolutionary function and origin of vertebrate traits.
